# A study on residue levels of fungicides and insecticides applied according to the program of raspberry protection

**DOI:** 10.1007/s11356-017-1098-4

**Published:** 2018-01-06

**Authors:** Stanisław Sadło, Bartosz Piechowicz, Magdalena Podbielska, Ewa Szpyrka

**Affiliations:** 1 Department of Analytical Chemistry, Institute of Biotechnology, University of Rzeszów, Pigonia 1 St, 35-310 Rzeszów, Poland; 2 Laboratory of Pesticide Residue Analysis, Regional Experimental Station in Rzeszów, Institute of Plant Protection – National Research Institute, Langiewicza 28 St, 35-101 Rzeszów, Poland

**Keywords:** Dietary intake, Fungicide, Insecticide, Maximum residue levels, Raspberry (*Rubus idaeus* L.), Residue levels

## Abstract

This paper presents surveys on residue levels of fungicides and insecticides applied according to the raspberry protection program. The field trials were conducted in 2013–2014 on a plantation of raspberry of the Laszka variety dessert raspberry very popular in Poland. Laboratory samples were collected starting from a day of the first fruit picking to the end of harvest. The highest mean residue levels were found for boscalid and pyraclostrobin (2.395 mg/kg and 0.732 mg/kg, respectively), in both cases they were at a level of about 24% of their maximum residue levels (MRLs); and for cypermethrin (0.235 mg/kg; i.e. close to 50% of its MRL). The long-term dietary intakes of those substances by Polish adult consumers were also at low levels of 0.52, 0.22, and 0.04% of acceptable daily intake (ADI), respectively. Therefore, the results obtained indicated that even on day zero of picking ripe raspberries, the pesticide residues not only were well below their corresponding MRLs, but also their daily intakes did not even approach 1% of the ADI. In 2013, pesticide residues in ripe fruit evolved according to a pattern different than in a subsequent year; while in 2014 they changed at a constant exponential rate.

## Introduction

Raspberries belong to berry fruit most frequently and most eagerly consumed by adults and children in Poland (Bobinaité et al. 2012) and frequently are eaten fresh without wash or processing. Our domestic production satisfies 20.1% of the global demand (Dmochowska 2012). Both leaves and roots of this plant are used in the production of tea, infusions and pharmaceuticals (Ryan et al. 2001; Holst et al. 2009). However, like some other crops, the raspberry plantations require a precise protection against pests and diseases (Graham and Jennings 2009; Mochecki 2014).

### Raspberry pests and diseases

Raspberries are susceptible to pests and to diseases caused by various fungal infections. A serious problem in Poland are also soil pests, especially grubs, including larvae of the widely widespread herbivorous May bug (*Melolontha melolontha* L.), a beetle from the *Scarabaeidae* family (Milenkovic and Stanisavljevic 2003; Totic 2014; Skrzecz et al. 2014; Sukovata et al. 2015). Other insects affecting raspberry crops are the raspberry beetle (*Byturus tomentosus* De Geer), the two-spotted spider mite (*Tetranychus urticae* Koch), and the blossom weevil (*Anthonomus rubi* Herbst). The main raspberry diseases are grey mould (*Botrytis cinerea* Pers.), and antracnose (*Gloeosporium Venetum* Speg). Control of diseases of fungal origin requires a particular care and some knowledge about their sources. Unlike pests and weeds (which, generally, appear over one or two periods), pathogenic fungi may occur several times during the growing season. They also cause serious problems during store and transport of ripe fruit (Jennings 1982; Fox 2006; Faby 2008; O’Neill et al. 2012), as they change the taste and the appearance of raspberries, and they may also be a source of mycotoxins (Moss 2008; Mitchell et al. 2010). In those cases the protective effect is ensured by deposits of plant protection products (PPPs) present on the plant surface, mainly on leaves and fruit at a moment of possible infection. However, presence of residues of their active ingredients (AIs), required for the crop protection, is harmful to the health of consumers (Nolan et al. 1984; Fenske et al. 1990; Lee et al. 2004; Piechowicz et al. 2012; Piechowicz 2013). Regular research proves that raspberries may contain different and, very frequently, high residue levels of fungicides and insecticides (Sadło et al. 2007; Słowik-Borowiec et al. 2012; Szpyrka et al. 2015).

### Pesticide use in raspberry protection

In Poland, systematic monitoring of the correctness of pesticide application in fruit and vegetables crops has been conducted since 1980s. Our first publications in this field concerned the determination of methidathion, deltamethrin and cypermethrin residues in hop cones, pesticide residues in greenhouse vegetables from south-eastern Poland, and the penetration of pesticides used in greenhouses to surface waters. The knowledge gathered during these surveys, as well as in the later period, forms a foundation for our opinion in this regard (Sadło 1990a, b; Sadło and Rupar 1990a, b; Szpyrka et al. 2008).

Recent studies have shown that nearly all raspberry samples contained residues of active ingredients of plant protection products, and quite frequently also so-called multiple residues. For example, in 2010–12, 42% of the surveyed samples of raspberries contained pesticide residues; moreover, 4% of them exceeded the MRL (Szpyrka et al. 2015). The situation was similar in other years (Sadło et al. 2007; Słowik-Borowiec et al. 2012). As apparent from other sources (Gnusowski et al. 2010), residues of some pesticides appeared also in fruit and leaves of raspberries produced using organic methods. All these observation imply there is a serious problem concerning the protection of raspberry plantations.

The supervised field trials conducted in 2013–2014 aimed at comprehensive studies on residues of fungicides and insecticides applied according to the raspberry protection program, determination of parameters of their exponential decomposition throughout the fruiting period, and the estimation of health risks for consumers. Additional aim was an estimation of the approximated relationship between the application rate and the residue level.

## Materials and methods

### Field trials

The supervised field trials were conducted at a commercial raspberry plantation (planted in 2012 and covering an area of 0.2 ha), which was protected against pests and diseases by plant protection products recommended in current programs (Table 1). The plantation was located in south-eastern Poland, in the village Grabówka Kolonia, in the province of Lublin and was planted on brown soil. On the raspberry plantation, four rows of plants were chosen for the study, each approximately 150 m long. On each sampling date, laboratory samples (each of them consisting of 32 ripe raspberries picked from randomly selected plants) were collected from each of the selected rows. Sampling dates covered the period from the first day of fruit picking (June 28, 2013, and June 17, 2014), up to the end of fruit bearing (July 25, 2013, and July 15, 2014). In total, 40 laboratory samples were analysed.

**Table 1:** Spraying history: treatments dates for plant protection products (PPP), their active ingredients (AI), application rates and pre-harvest intervals (PHI)

Treatments date	PPP, Trade name	AI, Common name	Application rate [kg/ha or L/ha]		PHI [day]
			PPP	AI	
2013					
May 15	Dursban 480 EC*	Chlorpyrifos	2.00	0.960	44
May 20	Folpan 80 WG	Folpet	1.00	0.800	39
May 24	Karate Zeon 050 SC	λ-cyhalothrin	0.30	0.015	35
June 1	Domark 100 EC	Tetraconazole	0.50	0.050	27
June 10	Mythos 300 SC	Pyrimethanil	2.50	0.750	18
June 17	Bellis 38 WG	Boscalid	1.50	0.378	11
		Pyraclostrobin	1.50	0.192	11
June 21	Pirimor 500 WG	Pirimicarb	0.75	0.375	7
June 24	Cyperkill Super 250 EC	Cypermethrin	0.15	0.038	4
June 25	Unix 70 WG	Cyprodinil	0.50	0.350	3
June 28	Signum 33 WG	Boscalid	1.80	0.481	0
		Pyraclostrobin	1.80	0.121	0
June 28	Date of first sampling/raspberry picking				
2014					
May 14	Dursban 480 EC*	Chlorpyrifos	2.00	0.960	34
	Cyperkil Super 250 EC	Cypermethrin	0.15	0.038	34
June 14	Mythos 300 SC	Pyrimethanil	2.50	0.750	29
June 23	Amistar 250 SC	Azoxystrobin	0.50	0.125	25
	Score 250 SC	Difenoconazole	0.50	0.125	25
June 29	Cyperkil Super 250 EC	Cypermethrin	0.15	0.038	19
June 30	Signum 33 WG	Boscalid	1.80	0.481	18
		Pyraclostrobin	1.80	0.121	18
July 4	Mythos 300 SC	Pyrimethanil	2.50	0.750	13
June 17	Date of first sampling/raspberry picking				

PPP, plant protection product; AI, active ingredient

PHI (PreHarvest interval)—the time between the last application of a given PPP and harvesting of the treated crops

*Product applied to soil

Plant protection products (PPPs; see Table 1) were applied according to directions in their respective labels, which can be found on the website of the Ministry of Agriculture of Poland (Wyszukiwarka Środków Ochrony Roślin 2017).

In both years, laboratory samples were collected on five sampling dates, starting from the day of the first picking of ripe raspberries to the end of fruiting of the plantation, and then residues of folpet, tetraconazole, pyrimethanil, boscalid, pyraclostrobin, cyprodinil, azoxystrobin and difenoconazole (fungicides) as well of chlorpyrifos, λ-cyhalothrin, pirimicarb and cypermethrin (insecticides) were determined.

Chlorpyrifos, an organophosphorus insecticide (OP), is an AI in Dursban 480 EC, used against *Melolontha melolontha* L., *Otiorhynchus* sp. and *Elateriadae* sp. threatening raspberry plantations (Clark et al. 2012; Piechowicz et al. 2015). Dursban 480 EC acts by contact, ingestion and inhalation. In 2013 and 2014, this insecticide was applied to the soil at a dose of 2 l per hectare (0.96 kg of chlorpyrifos per hectare), 44 and 34 days before the first crop picking, respectively.

Signum 33 WG (AIs: boscalid—a systemic compound from the anilides group, and pyraclostrobin—belonging to the strobilurin fungicides) is a plant protection product marketed in a form of water dispersible granules (WG) for preparing suspension of the working solution used for spraying raspberry plantations to control grey mould and raspberry spur blight, caused by *Didymella applanata* (Niessl), *Leptosphaeria coniothyrium* (Sacc.), *Botrytis cinerea* (Pers.) and *Elsinoe veneta* (Burkh.). As reported in Table 1, this fungicide was applied on June 28, 2013 (PHI: 0), and on June 30, 2014 (PHI; 18) at an application rate of 1.8 kg/ha, corresponding precisely to 0.4806 kg of boscalid and 0.1206 kg of pyraclostrobin per hectare. Moreover, Bellis 38 WG (AIs: 25.2% *w*/*w* boscalid and 12.8% *w*/*w* pyraclostrobin) was applied on June 17, 2013 (PHI: 11).

Mythos 300 SC (AI: pyrimethanil, a compound from the anilino-pyrimidines group) is a contact acting fungicide for preventive and interventional application to protect the raspberry plantation against grey mould caused by *Botrytis cinerea* (Pers.) (O’Neill et al. 2012), and withering of shoots caused by *Didymella applanata* (Niessl), *Leptosphaeria coniothyrium* (Sacc.)*, Botrytis cinerea* (Pers.)*, Verticillium* spp*.* and *Fusarium* spp. (Jennings 1982; Fox 2006; Faby 2008). This PPP was applied on June 10, 2013 (PHI: 18 days), June 14 (PHI: 29 days), and July 4, 2014 (PHI: 13 days), at a dose of 0.75 kg of pyrimethanil per 1 ha, in all cases.

Unix 70 WG (AI: cyprodinil, a compound of anilino-pyrimidine group) was used only once, on June 25, 2013, i.e., 3 days before the first raspberry picking, at a dose of 0.5 kg per hectare (0.35 kg of the AI per hectare).

Folpan 80 WG (AI: folpet, a contact acting compound from the phthalimides group), Domark 100 EC (AI: tetraconazole, a compound from the azole group), Amistar 250 SC (AI: azoxystrobin, a compound from the strobilurines group), and Score 250 SC (AI: difenoconazole, a compound from the azole group) in the form of water dispersible granules (WG), emulsifiable concentrates (EC) and suspension concentrates (SC) were applied 39, 27, 25 and 25 days before the first picking, respectively.

Cyperkill Super 250 EC (AI: cypermethrin, a compound from the synthetic pyrethroids group) acts against insects by contact and by ingestion. When used at raspberry plantations, it controls *Anthonomus rubi* (Herbst) and aphids –*Amphorophora idaei* (Börn) and *Amphorophora rubi* (Kalt.), pests commonly threatening raspberry crops (Kovanci et al. 2005; Borowiak-Sobkowiak 2006; Moss 2008). Cyperkill Super 250 EC was applied only once in 2013 (PHI: 4 days), and twice in 2014 (PHI: 34 and 19 days), at a dose of 0.15 l per hectare (0.0375 kg of AI per hectare), before the first crop picking.

Pirimor 500 WG (AI: pirimicarb) is an insecticide used at the application rate of 0.75 L per hectare (0.375 kg of pirimicarb per hectare), 7 days before the first raspberry picking. Pirimicarb, a fast-acting, selective aphicide belonging to the methyl carbamates group, is useful both against the organophosphate (OP)-resistant, and non-OP-resistant strains. It acts by contact, translaminar, by vapour, and has a systemic effect. It is used to protect a wide range of crops, including cereals, sugar beet, potatoes, fruit, and vegetables. It is relatively nontoxic to beneficial predators, parasites, and bees.

### Extraction procedure

Analytical portions (consisting of 16 ripe raspberries) collected from the laboratory samples were homogenised in a blender (Waring Commercial 8010 EG) with 150 mL of acetone, and then the obtained homogenates were filtered (MN 640 DD) on a Büchner funnel under vacuum. The blender jar was flushed with 50 mL acetone, and the washings were used to wash the filter cake. 1/5 the volume (the equivalent of approx. 15 g of fruit) of the obtained filtrate was taken for the further analysis, and was placed in a separatory funnel together with 100 ml of 2.5% sodium sulphate(VI) solution. Pesticide residues were extracted three times using, successively, 20, 10 and 10 ml of dichloromethane. The combined extracts were evaporated to dryness, dissolved in approx. 10 mL of petroleum ether, and purified on a Florisil mini-column (Valverde-Garcia et al. 1993). Pesticides were eluted with 70 mL of the ethyl ether-petroleum ether 3:7 (*v*/*v*) mixture, followed by 70 mL of the acetone-petroleum ether 1:9 (*v*/*v*) mixture (Sadło et al. 2014; Sadło et al. 2015).

### Chromatographic analysis

The final extracts were analysed by the Agilent 7890 gas chromatograph, equipped with the electron capture detector (μECD) and the nitrogen-phosphorus detector (NPD), and the fused silica column (HP-5 MS Ultra Inert column, 30-m length, 0.32-mm i.d., and 0.25-μm film thickness), operating in the splitless injection mode, connected to the NP (nitrogen-phosphorus) and EC (electron capture) detectors using a universal Y-splitter. The temperature of the injector and the detectors was 250 °C and 300 °C, respectively. The oven temperature was programmed as follows: 100 °C - 0 min → 10 °C/min → 180 °C - 4 min → 3 °C/min → 220 °C - 15 min → 10 °C/min → 260 °C - 11 min; the total time of the analysis was 55.3 min. Nitrogen (purity 6.0, flow 4.14 mL/min) was the carrier gas and the makeup gas for the μECD (30 mL/min) and NPD (10 mL/min). For the NPD, hydrogen and air flows were kept at 3 mL/min and 60 mL/min, respectively.

The recovery studies were conducted by spiking analytical portions of raspberries with stock solutions of pesticides. The average recoveries of folpet, tetraconazole, pyrimethanil, boscalid, pyraclostrobin, cyprodinil, azoxystrobin and difenoconazole (fungicides) and of chlorpyrifos, λ-cyhalothrin, pirimicarb and cypermethrin (insecticides) were within the range of 85.7 to 118.1%, with the relative standard deviations (RSDs) not exceeding 10.7% (for pyraclostrobin). The estimated limits of their quantification from ripe raspberries were 0.002 mg/kg, and the limits of detection were 0.001 mg/kg. In addition to the in-house quality assurance programme, the Laboratory of Pesticide Residue Analysis of the Institute of Plant Protection successfully participated in international proficiency testing schemes confirmed by certificates.

### Data analysis

The content/residue level (R_i_) of each substance in ripe raspberries was expressed as mg/kg. The mean values (R_M_), as well as the total residue levels of all substances (Tables 2 and 3) found in the four samples collected on each sampling date were calculated according to Eq. 1:

1 $$ {R}_M=\frac{1}{n}{\Sigma}_{i=1}^n{R}_i $$

**Table 2:** Presence of pesticide residues in ripe raspberries of the Laszka variety throughout a harvest period, 2013

Sampling date		Chlorpyrifos	Folpet	Tetraconazole	Pyrimethanil	Pyraclostrobin	Boscalid	Pirimicarb	Cypermethrin	Cyprodinil	Total
June 28	*R*_*M*_ [mg/kg]	0.002	0.003	0.013	0.182	0.732	2.395	0.369	0.111	0.314	4.121
	SD [mg/kg]	0.001	0.002	0.003	0.054	0.203	0.683	0.104	0.057	0.054	0.734
	%MRL	0.4	0.0	6.6	1.8	24.4	23.9	18.4	22.3	3.1	101.0
	%ADI	0.00	0.00	0.03	0.01	0.21	0.52	0.09	0.02	0.09	0.97
July 4	*R*_*M*_ [mg/kg]	0.002	0.006	0.004	0.010	0.088	0.236	0.050	0.009	0.138	0.545
	SD [mg/kg]	0.001	0.004	0.002	0.014	0.033	0.160	0.028	0.006	0.052	0.218
	%MRL	0.4	0.1	2.1	0.1	2.9	2.4	2.5	1.9	1.4	13.8
	%ADI	0.00	0.00	0.01	0.00	0.03	0.05	0.01	0.00	0.04	0.14
July 11	*R*_*M*_ [mg/kg]	0.002	0.002	0.002	0.006	0.124	0.456	0.095	0.012	0.042	0.740
	SD [mg/kg]	0.000	0.001	0.001	0.002	0.016	0.099	0.009	0.003	0.002	0.100
	%MRL	0.3	0.0	1.1	0.1	4.1	4.6	4.7	2.3	0.4	17.7
	%ADI	0.00	0.00	0.00	0.00	0.04	0.10	0.02	0.00	0.01	0.18
July 18	*R*_*M*_ [mg/kg]	0.002	0.004	0.004	0.014	0.164	0.484	0.062	0.008	0.031	0.773
	SD [mg/kg]	0.001	0.005	0.000	0.003	0.032	0.084	0.023	0.002	0.015	0.126
	%MRL	0.5	0.4	2.0	0.1	5.5	4.8	3.1	1.5	0.3	17.9
	%ADI	0.00	0.00	0.01	0.00	0.05	0.11	0.02	0.00	0.01	0.19
July 25	*R*_*M*_ [mg/kg]	0.002	0.007	0.004	0.004	0.168	0.457	0.066	0.004	0.008	0.719
	SD [mg/kg]	0.000	0.009	0.003	0.002	0.110	0.257	0.046	0.004	0.002	0.419
	%MRL	0.5	0.1	2.0	0.0	5.6	4.6	3.3	0.8	0.1	16.9
	%ADI	0.00	0.00	0.01	0.00	0.05	0.10	0.02	0.00	0.00	0.18

R_M_, mean residues; SD, standard deviation; MRL, maximum residue level; ADI, acceptable daily intake

**Table 3:** Presence of pesticide residues in ripe raspberries of the Laszka variety throughout a harvest period, 2014

Sampling date		Chlorpyrifos	Pyraclostrobin	Boscalid	Cypermethrin	Difenoconazole	Azoxystrobin	Pyrimethanil	Total
June 17	*R*_*M*_ [mg/kg]	0.002	0.047	1.196	0.235	0.016	0.006	0.148	1.650
	SD [mg/kg]	0.000	0.028	0.519	0.118	0.006	0.002	0.087	0.563
	%MRL	0.5	1.6	12.0	47.0	1.0	0.1	1.5	63.6
	%ADI	0.00	0.01	0.26	0.04	0.01	0.00	0.01	0.34
June 25	*R*_*M*_ [mg/kg]	0.002	0.030	0.744	0.123	0.009	0.003	0.035	0.949
	SD [mg/kg]	0.001	0.007	0.326	0.084	0.002	0.001	0.003	0.367
	%MRL	0.4	1.0	7.4	24.7	0.6	0.1	0.4	34.6
	%ADI	0.00	0.01	0.16	0.02	0.01	0.00	0.00	0.20
July 2	*R*_*M*_ [mg/kg]	0.001*	0.006	0.364	0.061	0.007	0.003	0.022	0.466
	SD [mg/kg]	0.000	0.007	0.135	0.033	0.001	0.001	0.004	0.123
	%MRL	0.3	0.2	3.6	12.3	0.5	0.1	0.2	17.2
	%ADI	0.00	0.00	0.08	0.01	0.01	0.00	0.00	0.10
July 8	*R*_*M*_ [mg/kg]	0.001*	0.005	0.205	0.021	0.008	0.003	0.011	0.256
	SD [mg/kg]	0.000	0.003	0.104	0.004	0.001	0.001	0.005	0.114
	%MRL	0.2	0.2	2.1	4.1	0.5	0.1	0.1	7.2
	%ADI	0.00	0.00	0.04	0.00	0.01	0.00	0.01	0.06
July 15	*R*_*M*_ [mg/kg]	0.001*	0.000*	0.046	0.018	0.008	0.004	0.007	0.083
	SD [mg/kg]	0.000	0.000	0.010	0.002	0.001	0.001	0.002	0.009
	%MRL	0.1	0.0	0.4	3.6	0.5	0.1	0.1	4.9
	%ADI	0.00	0.00	0.01	0.00	0.01	0.00	0.00	0.02

R_M_, mean residues; SD, standard deviation; MRL, maximum residue level; ADI, acceptable daily intake

*Residue level below limit of determination

Where *i* (1, 2, …, *n* = 4) is the sample number, and R_i_ represents the residue of a given substance, or the sum of residues of all substances, found in a given sample.

The residue of a given substance (R_i_) found in one of the four samples collected on a given sampling date, was divided by its respective maximum residue level (MRL), and then its mean percentage of the MRL was calculated using Eq. 2:

2 $$ \%\mathrm{MRL}=100{\Sigma}_{i=1}^n\frac{R_i}{MRL} $$

where *R*_i_ and MRL corresponded to the residue level of a given substance found in one of the four samples and to its legally accepted MRL in force in Poland in 2013–2014, respectively. The values of %MRLs of all substances (so-called multiple residues) found in each of the four samples were summed up and then their total mean percentages of the respective MRL values were estimated.

Using the residue level of a given substance (R_i_), and assuming the body weight (b.w.) of 76 kg, as well as a daily consumption (C) of raspberry by an adult Polish consumer of 0.007 kg, the substance long-term dietary intake with ripe raspberries was calculated and expressed as %ADI (Acceptable Daily Intake) (FAO 2009; Sadło et al. 2015), and then the mean percentages of respective ADI values for each of the four samples collected at sampling days were calculated. Similarly, assuming additive impact of various pesticides on the human organism, the total long-term daily intakes (in %ADI) of all substances applied within the framework of plant protection program of the dessert raspberry plantation were calculated according to Eq. 3:

3 $$ \%\mathrm{ADI}=100\frac{C}{b.w.}{\Sigma}_{i=1}^n\frac{R_i}{ADI} $$

Finally, based on the calculated long-term daily intake of a given substance (R_i_) with ripe raspberries expressed as %ADI, and a daily raspberry consumption (C = 0.007 kg) by an adult Polish consumer, the safe consumption level of the fruit (C_safe_ in kg) could be easily calculated using Eq. 4:

4 $$ {\mathrm{C}}_{\mathrm{safe}}=100\frac{C}{\% ADI} $$

Decreasing trends for the mean residue levels expressed as mg/kg and in %MRL, as well as their daily intakes expressed as %ADI were described by an exponential equation (Table 2) and by Figs. 3 and 4:

5 $$ {\mathrm{R}}_{\mathrm{t}}={R}_0\times {e}^{- kt} $$

where R_0_ and R_t_ are the mean residue levels (also %MRL or %ADI) of a given substance on day 0 and day *t* after the first picking of ripe raspberries, respectively. MRL and ADI values were taken from the European Union Pesticides Database website (European Union Pesticides Database 2017).

### Rainfall analysis

**Fig. 1:**
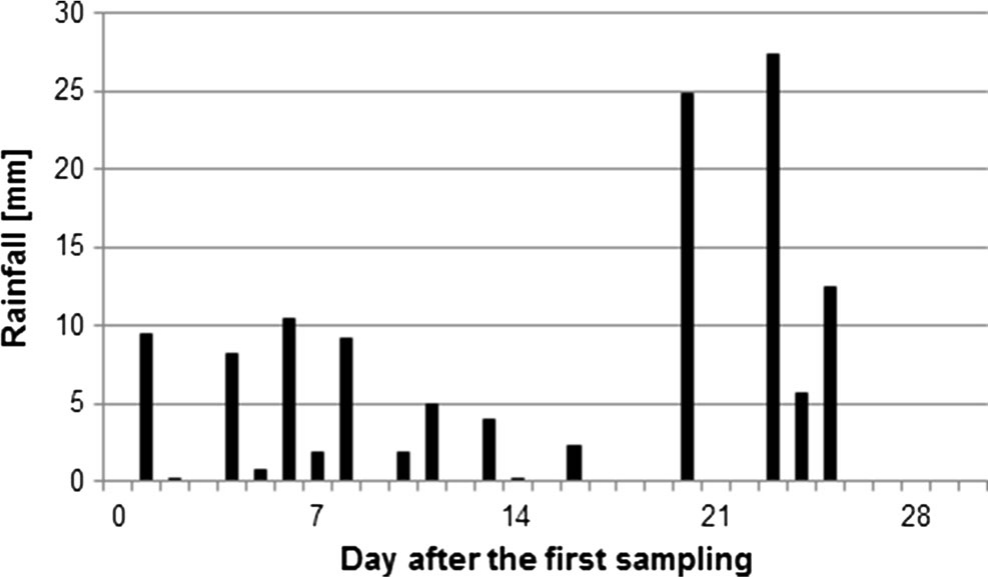
Daily rainfall throughout the harvest period; 2013

**Fig. 2:**
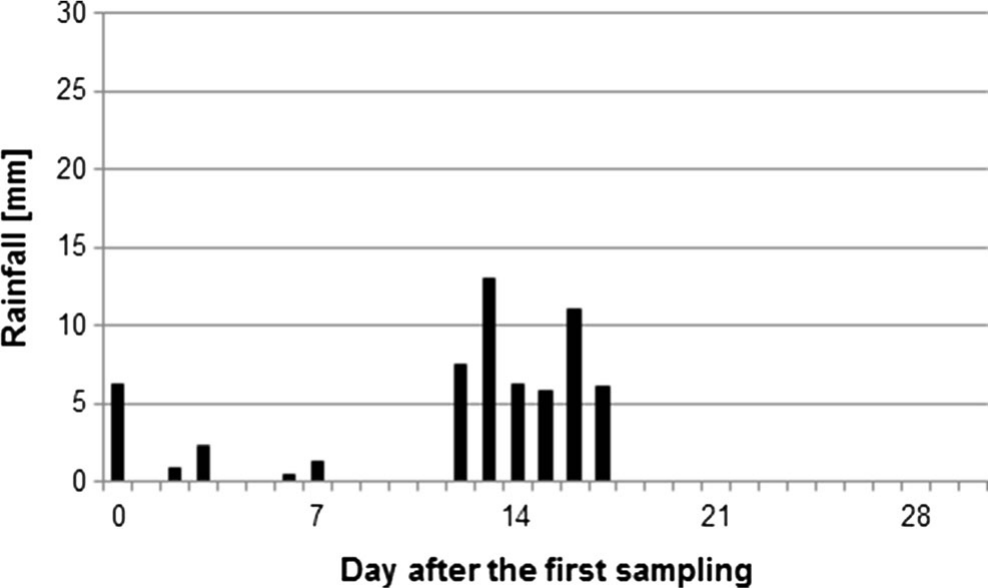
Daily rainfall throughout the harvest period; 2014

Rainfall measurements were performed using a mobile weather station WatchDog 2900ET (Spectrum Technologies, Inc. USA) located about 100 m from the raspberry plantation (Figs. 1 and 2).

## Results and discussion

### Weather conditions

The daily rainfall was measured throughout the harvest periods of harvest in 2013 and 2014 (Figs. 1 and 2, respectively). The rainfall has impact on boscalid and cypermethrin behaviour and, therefore, was considered in the discussion of results of these AIs. In the case of others AIs, there was no observed influence of rainfall on their dissipation rate.

### Fungicide residues in ripe raspberries

The mean levels (R_M_) with relevant standard deviations (SD) were calculated for all AIs (fungicides: folpet, tetraconazole, pyrimethanil, boscalid, pyraclostrobin, cyprodinil, azoxystrobin and difenoconazole as well of insecticides: chlorpyrifos, λ-cyhalothrin, pirimicarb and cypermethrin) in ripe raspberries and are presented in Tables 2 and 3.

#### Boscalid and pyraclostrobin residues

In consequence of Bellis 38 WG (2013) and Signum 33 WG (2013 and 2014) treatments, on day zero of the ripe raspberries harvesting, the mean residues of boscalid amounted to 2.395 mg/kg (2013; 24.4% of MRL) and 1.196 mg/kg (2014; 12.0% of MRL), and were the highest of all ingredients. High levels of boscalid residues in ripe raspberries, especially in 2013, were not only a consequence of a relatively high application rate of Signum 33 WG, applied just before the first raspberry picking (PHI: 0), and the differences between its levels deposited on ripe raspberriesin 2013 and 2014 (Table 4), but also resulted from application of Bellis 38 WG 11 days before the first harvest.

**Table 4:** Parameters of exponential disappearance trends for some pesticides used on a raspberry plantation in 2013–2014

Pesticide	R_0_ [mg/kg]	k [day]	R^2^	t_R = 0.01_ [day]	R_t = 7_ [mg/kg]	t_1/2_ = ln2/k [day]	MRL [mg/kg]
Boscalid, 2013	0.948	0.037	0.2276	n.d.	n.d.	n.d.	10
Boscalid, 2014	1.504	0.112	0.9458	45	0.687	6.19	10
Pyraclostrobin, 2013	0.296	0.033	0.2070	n.d.	n.d.	n.d.	3
Pyraclostrobin, 2014	0.050	0.123	0.9053	13	0.021	5.64	3
Cyprodinil, 2013	0.314	0.126	0.9758	27	0.130	5.50	10
Pyrimethanil, 2013	0.064	0.107	0.6144	17	0.030	6.48	10
Pyrimethanil, 2014	0.103	0.102	0.9392	23	0.051	6.80	10
Pirimicarb, 2013	0.179	0.046	0.4086	n.d.	n.d.	n.d.	2
Cypermethrin, 2013	0.051	0.097	0.7275	17	0.026	7.15	0.5
Cypermethrin, 2014	0.232	0.099	0.9649	32	0.118	7.00	0.5
Total residue of all AI, 2013	1.769	0.044	0.3460	n.d.	n.d.	n.d.	–
Total residue of all AI, 2014	1.976	0.105	0.9741	50	0.948	6.6	–

R_0_, initial pesticide concentration; k, first-order constant rate; *R*^2^, coefficient of determination; t_R = 0.01_, the time that must elapse until its residues reach the concentration level of 0.01 mg/kg; R_t = 7_, pesticide concentration after 7 days; t_1/2_, half-life time; MRL, maximum residue level; n.d., not determined, due to lack of any correlation (poor fit), the parameters of exponential disappearance have not been determined

In 2013, the boscalid residue levels found in ripe raspberry fruit evolved atypically. After their significant reduction, from 2.395 (the first sampling date) to 0.236 mg/kg (the second sampling date) during the first week of harvest, the mean residues remained at a similar level until the end of fruiting. The observed initial decline may be attributed to the rainfall, which occurred on July 29 and on August 1 and 3 (Fig. 1), disrupting an exponential (*R*^2^ = 0.2276) decreasing trend for residue levels.

On the other hand, in 2014, boscalid residues in ripe raspberry fruit decreased according to the exponential equation, R_t_ = 1.504e^-0.112t^ (*R*^2^ = 0.9458), with a half-life slightly below 7 days, and in laboratory samples, collected during the last week of fruiting, were found to be at the mean level of 0.046 mg/kg, which was still significantly above the standard for baby food of 0.01 mg/kg (Table 4).

**Table 5:** The mean mass of a single ripe raspberry throughout a harvest period; 2012–2014

Sampling term	2012*		2013		2014	
	Sampling date	Mean mass [g]	Sampling date	Mean mass [g]	Sampling date	Mean mass [g]
No. 1	June 19	5.3	June 28	5.3	June 17	6.1
No. 2	June 26	5.9	July 4	4.4	June 25	6.3
No. 3	July 3	4.1	July 11	5.6	July 2	6.5
No. 4	July 10	4.8	July 18	3.9	July 8	5.8
No. 5	July 17	3.8	July 25	4.2	July 15	5.4

*(Sadło et al. 2014)

In 2013 and in 2014, on first days of raspberry picking, the mean pyraclostrobin residue levels (the other AI of Signum 33 WG and of Bellis 38 WG) amounted to 0.732 mg/kg (about 24.4% of its MRL) and 0.047 mg/kg (no more than 2% of the MRL), respectively. Similarly as in the case of boscalid residues, in 2013 initial pyraclostrobin residues were decreasing atypically, while in 2014 their decrease could be described by the exponential curve, (*R*^2^ = 0. 9053), where *R*_0_ = 0.0501, and *k* = 0.123. It proves that the mean pyraclostrobin residue levels dropped by half within the first 6 days of crop picking, and the level of 0.01 mg/kg (10% of the beginning amount) would have been achieved within 13 days.

Boscalid was studied by Chen and Zhang (2010) in strawberries, where it was applied at two dosages (349.5 and 525.0 g AI/ha). The results showed that boscalid dissipation pattern followed the first-order kinetics with the half-lives of 4.9 and 6.4 days, respectively. The studies conducted by Jankowska et al. (2016) showed that the dissipation time for boscalid in tomatoes is 2.88 days for Marissa variety and 3.09 days for Harzfeuer variety. For the pyraclostrobin the dissipation time is 2.70 and 2.78 days for Marissa and Harzfeuer varieties, respectively.

#### Pyrimethanil residues

In 2013 and 2014, on day zero of raspberry picking, the mean pyrimethanil residues were at a level of 0.182 (PHI: 18 days) and 0.148 mg/kg (PHI: 13 days), respectively; thus, they were slightly below 2% of MRL. In both years, residue levels decreased significantly within the first weeks of the harvest. The exponential decline of the pyrimethanil residues in ripe fruit indicates clearly that their initial level dropped by half within 6–7 days and reached the level of 0.01 mg/kg after 17 and 23 days, respectively (see Table 4).

Szpyrka and Walorczyk (2013) reported that the dissipation time of pyrimethanil in different varieties of apples ranged from 11 to 22 days. Angioni et al. (2006) showed a half-life of 12 days for table grapes and 4.8 days for strawberries (Angioni et al. 2004).

#### Cyprodinil residues

Unix 70 WG (AI: cyprodinil) was applied in 2013. On day zero of raspberry picking, the mean cyprodinil residue was found to be at a level of 0.314 mg/kg (slightly above 3% of MRL). This fungicide disappeared according to the exponential equation, R_t_ = 0.3143e^-0.126t^, and thus its mean residue levels decreased by half in about 5–6 days. Despite its fast disappearance (*k* = 0.126), the mean residue level of 0.01 mg/kg may be reached no earlier than in 4 weeks.

The dissipation half-life of cyprodinil on and in various plants is within the range of 2.3–16.7 days (PPDB 2017). The half-life of cyprodinil was 9.6–20.8 days (Zhang et al. 2015) in grapes, and in strawberries it reached 14.5 days under the field conditions- and 5.5 days under greenhouse conditions (Liu et al. 2011). In our earlier study on the disappearance of cyprodinil residues on tomato leaves, its half-life was 9 days (Szpyrka and Sadło 2009).

#### Folpet, tetraconazole, azoxystrobin and difenoconazole residues

Folpan 80 WG (AI: folpet), Domark 100 EC (AI: tetraconazole), Amistar 250 SC (AI: azoxystrobin) and Score 250 SC (AI: difenoconazole) were applied 39, 27, 25 and 25 days before the first picking, respectively. Such prolonged PHIs, regardless of their application rates, generated only some trace residues of folpet, tetraconazole, azoxystrobin and difenoconazole in fruit, in the amount not exceeding 0.02 mg/kg just at the beginning of crop picking, so in practice, throughout the harvest, the ripe raspberries complied strictly to the standard for baby food of 0.01 mg/kg.

### Insecticide residues in ripe raspberries

#### Cypermethrin residues

Cyperkill Super 250 EC was applied only once in 2013 and twice in 2014. In 2013 and 2014, mean cypermethrin residues found on raspberries on first days of harvesting were at relatively high levels of 0.111 and 0.235 mg/kg (slightly above 22% and close to 47% of MRL, respectively). In 2013, those initial levels dropped significantly during the first week of the harvest (probably due to the rainfalls mentioned above), while in 2014 the residue level decreased strictly according to the exponential trend (*R*^2^ = 0.9649), and also dropped by half within 7 days, but during the whole harvesting period it remained at the level above 0.01 mg/kg (in 2014, there was only a slight rainfall; Fig. 2).

The dissipation half-life of cypermethrin on and in various plant is within the range of 1.2–10.3 days (PPDB 2017). In our study, this value in raspberry fruits was equal to 7.15 days in 2013 and 7.00 days in 2014 (Table 4). In our earlier study, half-lives of cypermethrin in raspberry fruits and leaves were 8 and 16 days, respectively (Sadło et al. 2014).

#### Pirimicarb residues

In 2013, Pirimor 500 WG (AI: pirimicarb) was applied 7 days before the first raspberry picking. The mean pirimicarb residue in ripe raspberry fruit was found to be at a relatively high level of 0.369 mg/kg, corresponding to 18.4% of MRL. Within the first week of fruiting, its residue dropped significantly, and, on average, was equal to 0.05 mg/kg. However, despite its initial significant decrease, the final residue level was still significantly above 0.01 mg/kg.

With its half-life of 4.9 days (PPDB 2017), pirimicarb is not persistent on a plant. In a study on its disappearance in apples, its half-lives were equal to 4 days and 11 days on leaves and fruits, respectively (Machowska and Sadło 2009).

#### Chlorpyrifos residues

In 2013 and 2014, the insecticide Dursban 480 EC (AI: chlorpyrifos) was applied to the brown soil 44 and 34 days before the first crop picking, respectively.

In our earlier studies conducted in 2012, residues of this compound were still detected at the level of 0.007 ± 0.0007 mg/kg in soil samples collected 6 weeks after the treatment (Sadło et al. 2014). In 2014, immediately after the treatment, the mean chlorpyrifos residue level in the 0–20 cm soil layer was 0.12 mg/kg, dissipating in accordance with the exponential equation, R_t_ = 0.116e^-0.052t^ (*R*^2^ = 0.878), and during the last week of the pre-harvest interval (PHI: 33 days) it was still at the level of 0.004 mg/kg. Chlorpyrifos half-lives in soils vary within the range of 1–100 days (Fang et al. 2006). Additionally, the low water solubility (1.4 mg L^−1^) and high log K_ow_ (4.7) values of this AI may result in insignificant mobility in soil (Min et al. 2009; MacBean 2012; IUPAC 2017). Therefore, it is likely that chlorpyrifos residues in arable soils are exposed to plant uptake (Hwang et al. 2017).

Throughout the period of harvesting, chlorpyrifos residues in ripe raspberries did not exceed 0.01 mg/kg, similarly as in 2012 (Sadło et al. 2015). Therefore, not only they were well below MRL (0.5 mg/kg) established for raspberry intended for adults, but also significantly below the level of 0.01 mg/kg, established for baby food.

### Total pesticide residues (sum of fungicide and insecticide residues) in ripe raspberries

In 2013, ripe raspberries collected for the analysis on day zero of the picking contained residues of eight AIs of PPPs (multiple residues). Their mean total content was found to be at a relatively high level of 4.121 ± 0.734 mg/kg, which, according to Eq. 1, corresponded to 101.0% of the respective MRLs, and was about two times higher than those found in 2014 (see Table 2) and 2012 (Sadło et al. 2014; Sadło et al. 2015). The total content of the compounds found in the samples collected on subsequent sampling dates was significantly lower, and their residues decreased at similar rates as observed for boscalid, pyraclostrobin, cypermethrin or pyrimethanil, although their final mean residues still amounted to 0.719 mg/kg, i.e. significantly above 0.01 mg/kg.

**Fig. 3:**
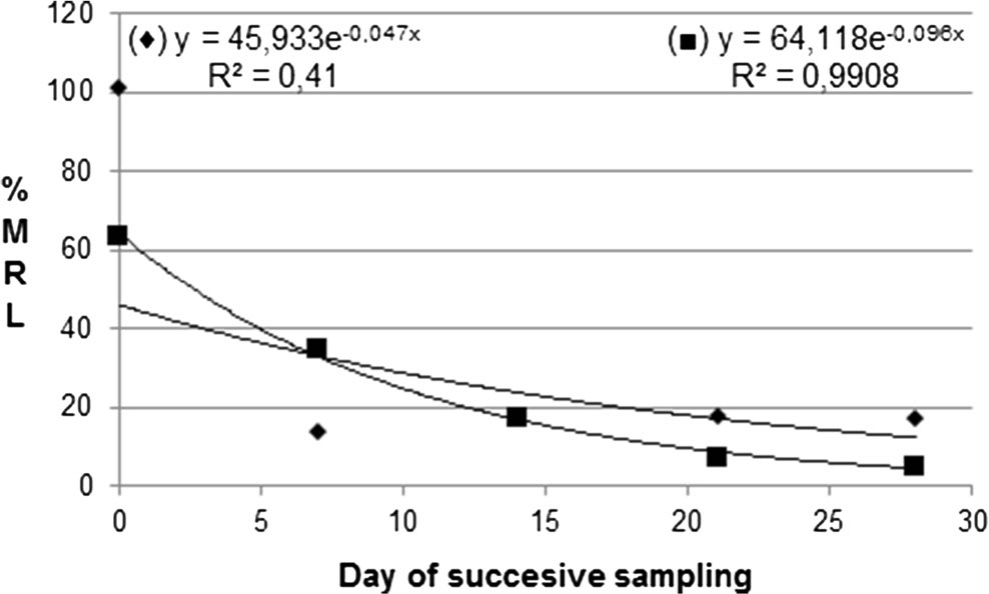
Exponential disappearances of total residues expressed as %MRL (maximum residue levels) throughout the harvest period in 2013 (♦) and 2014 (■)

In 2014, on the day zero of the picking, raspberry samples contained seven AIs of PPPs but their total residues were significantly lower than those found a year before (Table 3), and reached the mean level of 1.650 ± 0.563 mg/kg, corresponding to only 63.6% of MRL. In the subsequent weeks of the plantation fruiting, the total residues decreased exponentially (R_t_ = 64.118e^-0.096t^, *R*^2^ = 0.9908) (Fig. 3), and finally reached the level of 0.083 mg/kg, also significantly exceeding 0.01 mg/kg.

### Pesticide residues in ripe raspberries vs. pesticide application rates

The estimation of a relationship between the pesticide application rate and the residue level is not easy for several reasons. First, the treatments were performed when the fruit was at various stages of maturity. A clear correlation between a radius, a surface area and a volume of an individual raspberry fruit indicates that, for the same application rate of the active ingredient, smaller fruit should contain higher residue levels, when they are expressed as mg/kg. Second, the same PPPs were applied at different PHIs and, therefore, their deposits were subject to the influence of different weather conditions. Third, with the development of the bushes, not only the area of leaves (the canopy impact) increases, but also an area and properties of fruit surface vary. Finally, very frequently the pesticide is applied repeatedly, as it was in 2013, when Signum 33 WG (PHI: 0 days) was applied after Bellis 38 WG application (PHI: 11 days), and thus, the mean boscalid residue resulted from both treatments.

Despite the above, using the mean levels of boscalid residues detected in 2013–2014, we approximately estimated a relationship between the level of residues and the application rate (D; dose). This relationship shows that immediately after the application of any substance at a dose of 1 kg/ha, its residue levels in ripe raspberries should be within the range of 2.4917 to 4.9896 mg/kg (Table 6), and the mean level should be approximately 3.6 mg/kg. This rough estimate can indicate that the current MRLs are significantly overestimated and consequently are not a good tool for assessing the correctness of actions conducted to protect a plantation.

**Table 6:** An approximate relationship between a dose of an active ingredient in Signum 33 WG and its residue in ripe raspberries on the example of boscalid residues detected in 2012–2014

Year	AI common name	D of AI [kg]	R [mg/kg]	Regression equation	PHI [day]
2012*	Boscalid	0.48	0.950	*R* = 1.9792 × D	6
2013	Boscalid	0.48	2.395	*R* = 4.9896 × D	0
2014	Boscalid	0.48	1.196	*R* = 2.4917 × D	18

AI, active ingredient; D of AI, dose of active ingredient; R, pesticide residue; PHI, PreHarvest Interval

*(Sadło et al. 2014)

### Long-term dietary intakes of pesticide residues with ripe raspberries

Generally, long-term dietary intakes are calculated by multiplying mean residues obtained from supervised field trials by an average daily consumption per capita estimated for each commodity and expressed as a percentage of the ADI (FAO 2009). When the intake exceeds (is equal to or above 100% of ADI) the ADI level, the result may be interpreted as giving rise to a health concern.

**Fig. 4:**
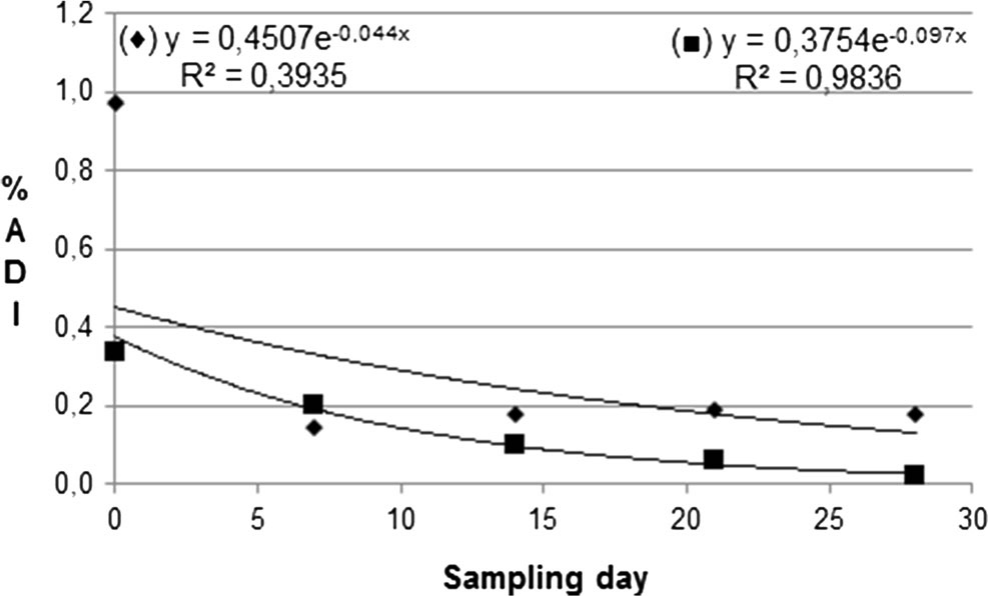
Exponential changes of the total long-term dietary intakes expressed as %ADI (acceptable daily intake) throughout the harvest period in 2013 (♦) and 2014 (■)

In our study, we decided to base our calculations on the individual pesticide residue levels found in a given sample, assuming body weight (b.w.) of 76 kg and a daily consumption of raspberries by an adult Polish consumer equal to 0.007 kg (C). Thus, to calculate the long-term dietary intake of a given substance (and its average level) with ripe fruit, four samples were collected for the analysis on each of five sampling dates. The highest daily intake was possible for boscalid (the average: 0.52% of its ADI), followed by pyraclostrobin (the average: 0.21% of its ADI) and pirimicarb (the average: 0.09% of its ADI) in 2013, and for boscalid (the average: 0.26% of its ADI), followed by cypermethrin (the average: 0.04% of its ADI) in 2014 (Fig. 4).

Moreover, assuming an additive effect of pesticide residues on a human body, the long-term daily intake of all substances (generating so-called multiple residues) applied within the framework of the plant protection program for a raspberry plantation, was calculated for each of four samples according to Eq. 2, and its mean values are shown in Tables 2 and 3. In 2013, the average long-term daily intake of all substances was 0.97% of their ADIs. During the first week, it decreased below 0.2% of the ADI and then remained at the constant level to the end of fruiting, while in 2014, the average long-term daily intake of all substances was 0.34% of their ADI, and then decreased exponentially (*R*^2^ = 0.9836), reaching its final value of 0.02% of ADI. Generally, in both years, the average long-term daily intakes were substantially below the acceptable daily intake and they should not cause any chronic health risk for an adult consumer.

### Safe daily consumption of ripe raspberries with residues of pesticides used in field trials in 2013–2014

Safe daily consumption of ripe raspberries can easily be calculated using Eq. 4. The highest total long-term dietary intake, equal to 0.97% ADI, indicates that, the safe level of raspberry consumption for an adult consumer exceeded 700 g a day over a lifetime, without any appreciable health risk in 2013, and exceeded even 2000 g in 2014. Moreover, taking into account that Acute Reference Dose (ARfD) for each of the studied substances is about 5 times higher than the respective Acceptable Daily Intake (ADI), the raspberry fruit from Poland is completely safe for the consumer.

## Conclusions

In 2013–2014, supervised field trials were conducted on commercial raspberry plantations. Obtained results clearly indicated that residues of the individual active ingredients of plant protection products in ripe fruit occurred at different levels, depending on an application rate and a PHI, i.e. the period that elapsed from the day of their application to day zero of fruit picking. In conclusion, therefore:

- the highest mean residue levels in ripe raspberries were found for boscalid and pyraclostrobin (2.395 and 0.732 mg/kg), in both cases they were at the level of about 24% of their MRLs, and for cypermethrin (0.235 mg/kg; i.e., close to 50% of its MRL),
- the long-term dietary intake of those substances by a Polish adult consumer was also at low levels of 0.52%, 0.22%, and 0.04% of ADI, respectively,
- the obtained results indicate that even on the day zero of fruiting the pesticide residues in ripe raspberries not only were well below the corresponding MRLs, but their daily intakes did not even approach 1% of the ADI,
- the residues of folpet, tetraconazole, azoxystrobin and difenoconazole, applied 39, 27, 25 and 25 days before the first fruit picking, respectively, residues of chlorpyrifos applied to the soil, and residues of pyraclostrobin, pyrimethanil and cypermethrin in the raspberry fruit picked in the third decade of harvest, were below or equal to 0.01 mg/kg,
- to produce the raspberries with the residue level below or equal to 0.01 mg/kg, it would be necessary to terminate the application of pesticides at least 2–3 weeks before the first crop picking and to ensure that a preparation efficient at low doses, for instance Domark 100 EC or Nissorun 050 EC, is applied as the last treatment,
- in 2013, pesticide residues in ripe fruit evolved according to a different pattern than in the subsequent year, while in 2014 they decreased exponentially at constant exponential rates ranging from 0.099 (cypermethrin) to 0.123 (pyraclostrobin).
